# Four New Vining Species of *Solanum* (Dulcamaroid Clade) from Montane Habitats in Tropical America

**DOI:** 10.1371/journal.pone.0010502

**Published:** 2010-05-05

**Authors:** Sandra Knapp

**Affiliations:** Department of Botany, The Natural History Museum, London, United Kingdom; Zoological Society of London, United Kingdom

## Abstract

**Background:**

*Solanum* (Solanaceae), with approximately 1500 species, is one of the largest genera of flowering plants, and has a centre of diversity in the New World tropics. The genus is divided into 13 major clades, of which two, the Dulcamaroid clade and the “African Non-Spiny” clade, exhibit vine morphology with twining petioles. I am currently preparing a worldwide monograph of these two groups, comprising some 70 species.

**Methods:**

I formally describe here four new species of *Solanum* from montane Mexico and South America all belonging to the Dulcamaroid clade (including the traditionally recognised section *Jasminosolanum* Bitter). Descriptions, discussions of closely related species and preliminary conservation assessments are provided for all species; all species are illustrated. This paper is also a test case for the electronic publication of new names in flowering plants.

**Conclusions:**

These new species are all relatively rare, but not currently of conservation concern. *Solanum aspersum* sp. nov. is distributed in Colombia and Ecuador, *S. luculentum* sp. nov. in Colombia and Venezuela, *S. sanchez-vegae* sp. nov. is endemic to northern Peru and *S. sousae* sp. nov. to southern Mexico. *Solanum luculentum* has the morphology of a dioecious species; this is the first report of this breeding system in the Dulcamaroid clade.

## Introduction

*Solanum* is one of the ten most species-rich genera of flowering plants [1]. With approximately 1500 species (J. Bennett & S. Knapp, unpubl.) occurring on all temperate and tropical continents, the genus occupies an incredibly wide range of habitats and habits, paralleling that of the family. The history of *Solanum* classification has been reviewed previously [2], but the last time the genus was monographed in its entirety was in De Candolle's *Prodromus* [3]. Current work by participants of the “PBI Solanum” project (see www.nhm.ac.uk/solanaceaesource) will result in a modern monographic treatment of the entire genus available on-line. *Solanum* can be divided into 13 well-supported monophyletic clades [4], [5], the largest of which is the group commonly known as the spiny solanums (the Leptostemonum clade). The largest non-spiny solanum clade is the Morelloid/Dulcamaroid clade, comprising all the herbaceous species previously placed in section *Solanum* [6] and allied groups, and the vining species placed in sections *Jasminosolanum*, *Dulcamara*., *Lysiphellos* and *Andropedas* plus a variety of other taxa [4]. All these species (plus other species previously recognised as section *Parasolanum* Bitter, e.g. *S. corymbosum* Jacq.) have previously been combined into a single section *Dulcamara* (Moench) Dumort. [6]; included in this circumscription the African *S. terminale* Forssk. and its relatives, which in molecular analyses form a distinct clade called the “African Non-Spiny” clade [4].

The monophyletic group here referred to as the Dulcamaroid clade appears to be sister to the Morelloids [5], and includes a wide variety of groups previously thought to be unrelated. The woody members of the *S. nitidum* species group [7] belong here, as do several shrubby species (e.g., *S. aligerum*, *S. pubigerum*) previously thought to allied to the members of section *Geminata* [2], [8]. Most members of the clade, however, are woody vines, often reaching the canopy and thus not often collected. Members of the clade share a peculiar inflorescence morphology in which the pedicel is inserted into a small cup or sleeve on the inflorescence axis, so that when abscission occurs a distinct cup is left behind [7]. The vining members of the group climb by means of twining petioles; petioles on older leaves are often very thick and woody.

Members of the Dulcamaroid group occur in both the Old and New Worlds, with the Old World taxa largely boreal (e.g., *S. dulcamara* L. and relatives) and those of the New World mostly tropical, with high species diversity in southeastern Brazil [9]. Montane regions are also areas of high species diversity in the group. In the course of preparing a monographic treatment of the Dulcamaroid group a number of new montane taxa were encountered; these are described here in order that the names may used in the on-line treatment and in floristic works. This contribution is the first to publish new plant names in a purely electronic journal, and thus serves as a test case for changes being developed for the International Code of Botanical Nomenclature [10]. Discussion of previous changes to the ICBN to facilitate electronic publication can be found elsewhere [11], [12], [13]. Digital images of all type specimens and complete specimen label information can be found on Solanaceae Source (www.nhm.ac.uk/solanaceaesource).

## Results and Discussion

### Taxonomic treatment

**Solanum aspersum** S.Knapp, sp. nov. [urn:lsid:ipni.org:names:77103633-1] Type: Colombia. Putumayo: vertiente oriental de la Cordillera, entre Sachamates y San Francisco de Sibundoy, 1600–1750 m, 30 Dec 1940, *J. Cuatrecasas 11471* (holotype, COL; isotypes, F [F-1335119], US [US-1799731]).

Figure 1.

**Figure 1:**
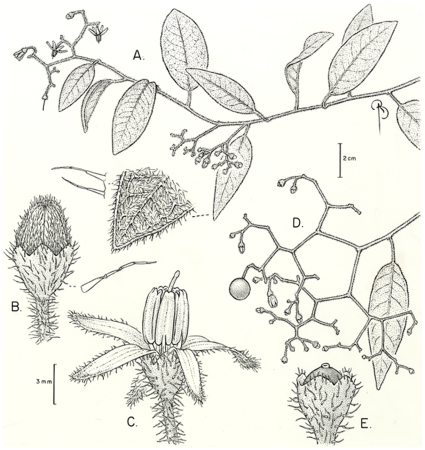
*Solanum aspersum*. A) portion of stem, with details of trichomes, B) young bud, C) open flower, D) fruiting inflorescence, E) young fruit. A–C drawn from *MacDougal et al. 4251*, D–E from *Zak 1857A*.

Species *Solano aureo* Dunal similis, sed foliis bullatis, trichomatibus uniseratis simplices, gemmis elongatis, corollis profunde stellatis, differt.

Woody vine, of unspecified length or height; stems densely and evenly pubescent with antrorsely curved simple uniseriate trichomes 0.5–1.5 mm long, these few-celled with a large basal cell, arising from expanded bases and eventually deciduous; new growth densely pubescent with simple uniseriate trichomes to 1.5 mm, these pale straw-colored in herbarium specimens; bark of older stems greenish brown, minutely tuberculate from the bases of the deciduous trichomes. Sympodial units plurifoliate. Leaves simple, (1−)3.5–9× (0.6−)1.5–4.6 cm, ovate to narrowly ovate, widest in the basal third, membranous or chartaceous, strongly discolorous, the upper surfaces evenly pubescent on veins and lamina, the trichomes to 2 mm long, simple, uniseriate, arising from expanded bases giving the lamina surface a tuberculate appearance, the lower surfaces densely and evenly pubescent with simple uniseriate trichomes to 2 mm, these 2-3-celled with the basal cell largest, denser on the veins; primary veins 7–9 pairs, impressed above in herbarium specimens; base truncate or shallowly cordate; margins entire, not revolute; apex acute to acuminate; petioles 0.7–2 cm, densely pubescent with simple trichomes like those of the stems and leaves. Inflorescences terminal on leafy short shoots, 3–15 cm long, globose to ellipsoid in outline, branching many times, with 2 principal basal branches, with 12–60 flowers, densely pubescent with simple trichomes; peduncle 0.5–3 cm, the branching very near the junction with the stem; pedicels 0.5–0.8 cm, <0.5 mm in diameter at the base and apex, pubescent with 1-2-celled simple trichomes to 1.5 mm long, spreading at anthesis, articulated near the base from a small sleeve, leaving a small peg on the axis; pedicel scars irregularly spaced 1–10 mm apart, the inflorescence rachis bending at almost right angles at articulation points. Buds narrowly ellipsoid, the corolla strongly exserted from the calyx tube. Flowers all perfect, 5-merous; calyx tube ca. 2 mm, conical, the lobes 0.5–1 mm, deltate to broadly deltate, pubescent with simple trichomes, these sparser than on the rest of the inflorescence; corolla 1.2–1.7 cm in diameter, white, pink or “pale blue” (violet?), narrowly stellate, lobed nearly to the base, the lobes 6–7×1.5–2 mm, reflexed at anthesis, glabrous adaxially, densely pubescent abaxially with weak simple papillate trichomes to 0.5 mm long, these denser on tips and margins; filament tube <0.5 mm, the free portion of the filaments ca. 0.5 mm, glabrous; anthers 4–4.5 x ca. 1 mm, yellow, ellipsoid, poricidal at the tips, the pores lengthening to slits with age; ovary glabrous; style 5–6 mm long, pubescent with weak simple trichomes to 0.5 mm, more densely pubescent in the basal half; stigma capitate-truncate, the surface minutely papillose. Fruit a globose berry, ca. 1.3 cm in diameter (immature?), green or yellowish green, the pericarp thin and shiny, glabrous; fruiting pedicels 0.9–1 cm, 1–1.5 mm in diameter at the base, woody and spreading. Seeds not seen from mature berries, apparently 10+ per berry and flattened reniform.

#### Distribution

*Solanum aspersum* occurs in widely separated and isolated populations along the Andes from central Ecuador into Colombia in both the Cordillera Occidental and the Cordillera Central, from 1600 to 2500 m.

#### Etymology

The specific epithet refers to the few and scattered collections of this species (aspersus  =  scattered) along the Andes from Ecuador to Colombia that have been subsumed in the more common *Solanum aureum*.

#### Preliminary conservation status

Although known from very few herbarium specimens, *S. aspersum* has a wide distribution, and is possibly more common in its total range than currently known.

#### Additional specimens examined

**Colombia**. **Antioquia**: Mun. Urrao, between Urrao and Caicedo, 21 km E of Urrao, near high point on road, 6°24′N, 76°02′W, 27 Feb 1989, *MacDougal et al. 4251* (MO). **Cundinamarca**: 17 Feb 1950, *von Sneidern 5825* (S). **Ecuador**. **Napo**: Parroquia Cosanga, 6 kms de la carretera Cosanga-El Aliso, 23 Aug 1990, *Jaramillo et al. 12110* (MO). Canton Quijos, Río Aliso, 8 km al suroeste de Cosanga, 0°37′S, 77°56′W, 15 Nov 1998, *Vargas et al. 3043* (MO). **Pichincha**: km 59 de la carretera antigua Quito-Santo Domingo de los Colorados, a 3.5 km al NE de la carretera, 28 Mar 1987, *Zak 1857A* (F, MO).

The few specimens of *Solanum aspersum* have usually been annotated as the more common and widely distributed *S. aureum* Dunal, also from Andean Ecuador. *Solanum aspersum* differs from that species in its simple uniseriate, rather than congested-dendritic pubescence (Fig 1A), and in the elongate buds that open to deeply stellate flowers (Fig. 1B, C). Specimens of *S. aureum* from Azuay province in Ecuador have similarly shiny adaxial leaf surfaces to *S. aspersum*, but always have the characteristic golden dendritic pubescence of that species rather than the simple pubescence of *S. aspersum*. The leaves of *S. aspersum* are usually more cordate than those of *S. aureum*, but some populations of *S. aureum* approach *S. aspersum* in overall leaf morphology at first glance. *Solanum aspersum* has a very scattered distribution all along the Andes from northern Colombia to central Ecuador and is likely to be found in more of the intervening parts of the cordilleras, but it is apparently rare and easily overlooked.

**Solanum luculentum** C.V.Morton ex S.Knapp, sp. nov. [urn:lsid:ipni.org:names:77103634-1] Type: Colombia. Antioquia: Mpio. Sonsón, Vereda Manzanares, Finca La Montañita, Cerro de la Vieja, páramo de Sonsón, 2600–3100 m, 11 Jan 1995, *J. Betancur & S.P. Churchill 5912* (holotype, COL [COL000057871]; isotype, HUA).

Figure 2.

**Figure 2:**
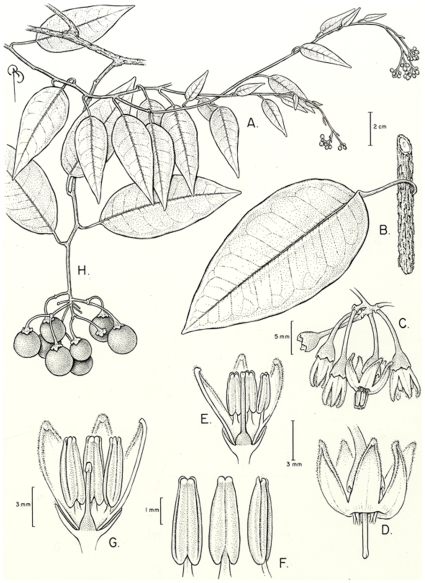
*Solanum luculentum*. A) portion of stem with flowering inflorescence, B) detail of stem with peeling bark, C) buds, D) open long-styled flower with anthers removed, E) long-styled flower cross-section, F) anthers, G) short-styled flower cross-section, H) fruiting inflorescence from putatively pistillate individual. A–B drawn from *Nee & Callejas 32546*, C–F from *Steyermark et al. 127855*, G from *Steyermark & Dunsterville 100777*, H from *Killip & Smith 15952*.

Species *Solano dichroandro* Dunal similis, sed corticibus exfoliatis, foliis glabris nitidis, floribus heterostylibus (unisexualibus?), differt.

Woody vines or lianas, occasionally apparently epiphytic, to 6 m; stems glabrous and shiny; new growth almost completely glabrous, with extremely sparse pubescence of minute, golden multiseriate trichomes <0.5 mm long; bark of older stems pale tan and markedly exfoliating (“shreddy” fide *Nee & Callejas 32546*). Sympodial units plurifoliate. Leaves simple, 2–11×0.l7–5 cm, elliptic to narrowly elliptic, coriaceous, the upper surfaces glabrous and shiny, the veins not apparent, the lower surfaces glabrous, the veins yellowish cream; primary veins 5–7 pairs, prominent below, obscure above; base cuneate to acute to truncate and occasionally slightly cordate; margins entire, strongly revolute in both dry and live (fide *Steyermark et al. 100777*) plants; apex acute or occasionally long acuminate; petioles 0.7–3 cm, glabrous or with a few scattered glandular papillae, wrinkly when dry. Inflorescences terminal, 3–11 cm long, more or less ellipsoid in outline, many times branched, with 20–50 flowers, glabrous; peduncle 0.5–2 cm long, branching from very near the base; pedicels 1.2–1.5 cm, slender, ca. 0.5 mm in diameter at the base, ca. 1 mm in diameter at the apex, glabrous, apparently somewhat erect at anthesis, articulated just above the base, leaving a prominent swelling on the axis; pedicel scars irregular spaced 2–10 mm apart. Buds globose, becoming ellipsoid to turbinate, the corolla strongly exserted from the calyx tube early in expansion. Flowers heterstylous, 5-merous, the plants probably dioecious, long-styled and short-styled flowers on different plants but of similar overall morphology; calyx tube 1.5–2 mm, conical, the lobes 0.5–1 mm, broadly deltate, glabrous with the tips minutely papillate; corolla 1.5–1.7 cm in diameter, white or occasionally tinged with lavender, stellate, lobed 2/3 to 3/4 of the way to the base, the lobes 6–7×3–4 mm, planar at anthesis, densely papillose on tips and margins with golden simple trichomes, these occasionally extending along the midvein of the abaxial surface; filament tube minute, the free portion of the filaments ca. 1 mm, glabrous; anthers of long-styled flowers ca. 4×1 mm, occasionally slightly shrivelled, those of short-styled flowers ca. 5×1.5 mm, yellow, ellipsoid to pointed ellipsoid, poridical at the tips, the pores with thickened margins and lengthening to slits with age; ovary glabrous, vestigial in short-styled flowers; style in long-styled flowers 5–6 mm, exserted beyond the anthers, glabrous, in short-styled flowers 2.5–3 mm, included in the anther tube, glabrous; stigma clavate, the surface densely papillose in long-styled flowers. Fruit a globose berry, to 2 cm in diameter, green (immature?), the pericarp quite thin but not markedly shiny; fruiting pedicels 1.5–2 cm, ca. 2–3 mm in diameter at the apex, woody and nodding. Seeds 10–12 per berry, 6–8×4–6 mm, flattened reniform, pale tan, the surfaces minutely pitted, the testal cells rectangular at the margins, deeply sinuate with rib-like thickenings on the lateral walls in the seed center.

#### Distribution

*Solanum luculentum* occurs in the Andes of Colombia (Depts. Antioquia, Cundinamarca and Nariño) and Venezuela (from the Colombian border to the Federal District around Caracas) in cloud forests from 1500 to 3200 m.

#### Etymology

The epithet, taken from annotations by the solanologist Conrad V. Morton on sheets in US, refers to the extremely shiny upper surfaces of the leaves (luculentus  =  full of light, splendid).

#### Preliminary conservation status

*Solanum luculentum* is widely distributed across northern South America and appears to be relatively common where it does occur, but the extent of damage to its cloud forest habitat needs assessment. It should be considered not threatened at present.

#### Additional specimens examined

**Colombia**. **Antioquia**: Mun. Caldas, Vereda La Corrala, al lado del camino al la cascada, 21 Sep 1987, *Albert de Escobar et al. 7939* (MO); sin. loc., 28 Dec 1930, *Archer 1153* (US,) 1 Jan 1931, *Archer 1227* (US); en los alrededores [de Medellín], 21 Aug 1948, *Barkley & Johnson 264* (US); near Medellín, *Bros. Daniel & Arsènio 3486* (US); La Ceja, 21 Jul 1944, *Bro. Daniel 3281* (US); midway between Medellín and Río Negro, 6°05′N, 75°25′W, 8 Jul 1986, *Nee & Callejas 32456* (US); Mun. Salgar, km 15 of road Salgar-Hacienda El Dauro (Dpto. Chocó), 5°59′N, 76°06′W, 14 Mar 1987, *Zarucchi & Echeverri 4753* (K); Mun. Jardín, km 20 of road Jardín-Riosucio (Dept. Caldas), ca. 15 km SSE of Jardín, 5°31′N, 75°48′W, 29 Oct 1988, *Zarucchi et al. 6928* (K). **Boyacá**: Cordillera Oriental, near Laguna Seca in valley of Río de los Pajaros, 26 Aug 1957, *Grubb et al. 737a* (K). **Cundinamarca**: carretera a Fusagasugá, 9 May 1949, *García-Barriga 13335* (US). **Santander**: in vicinity [of Santander], 21 Dec 1926, *Killip & Smith 15952* (US). **Venezuela**. **Aragua**: sin. loc., 1856, *Fendler 2099* (GOET, K, MO); 4 km SW by air, on road to Capachal 2 km east from road between Colonia Tovar and La Victoria, 10°22′N, 67°19′W, 7 Apr 1982, *Liesner & Medina 13496* (MO). **Distrito Federal**: Dept. Libertador, a lo largo del camino Costa de Maya, noroeste de la Colonia Tovar, 3–5 kms desde la carretera principal La Victoria-Colonia Tovar, 10°25′N, 67°20′05″W, 9 Dec 1982, *Steyermark et al. 127855* (MO). **Tachira**: cabeceras del Río Quinimari, entre el pié del peñasco de la Peña de Pata de Judio (debajo del páramo del Judio), y el pié del salto de Chorrejón de la Mota de la Peña de Ventana, arriba de Las Copas, 18–20 kms al sur de San Vicente de la Revancha, 32–35 kms al sur de Alquitrana, suroeste de Santa Ana, 12 Jan 1968, *Steyermark et al. 100777* (US).

*Solanum luculentum* was identified as a new species by the Solanaceae specialist ConradV. Morton in the 1940s on herbarium annotation slips on specimens in US (*Archer 1153*, *1227*), but the very appropriate name was never published. I have decided to use it here, as it perfectly describes the distinguishing characteristic of this species, its coriaceous, lustrous and shining leaves (Fig. 2B). *Solanum luculentum* has long been confused with *S. dichroandrum* Dunal, another vining species from northern South America, but differs from that in its completely glabrous leaves and inflorescences, revolute leaf margins and heterostylous flowers.

Specimens of *S. luculentum*, to my knowledge, either bear short-styled flowers and no fruits or long-styled flowers and fruits (see Fig. 2D, E, F); this is indicative of a dioecious species of *Solanum*, one of very few outside the Leptostemonum clade [14], and the first record for this breeding system in the Dulcamaroid clade. Field confirmation of the breeding system of *S. luculentum* will be interesting; pollen of this species has not yet been examined to ascertain if it is inaperturate, as is pollen of other dioecious solanums [14].

**Solanum sanchez-vegae** S.Knapp, sp. nov. [urn:lsid:ipni.org:names:77103635-1] Type: Peru. Amazonas: Prov. Chachapoyas, W side of Cerros Calla-Calla, 45 km above Balsas, mid-way on road to Leimebamba, 3100 m, 19 Jun 1964, *P.C. Hutchison & J.K. Wright 5738* (holotype, USM; isotypes, F [F-163831], K [K000545365], P [P00549320], US [US-246605], USM).

Figure 3, 4.

**Figure 3:**
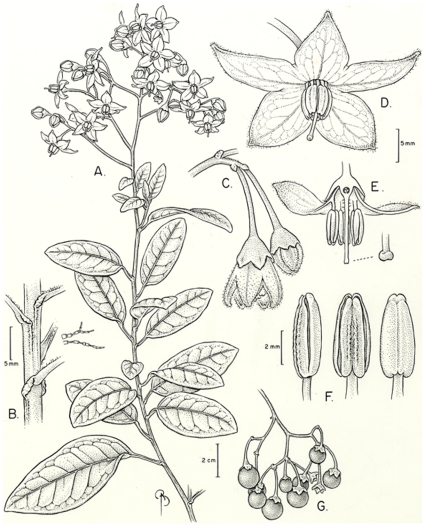
*Solanum sanchez-vegae*. A) portion of stem, B) petiole articulation, C) buds, D) open flower, E) flower cross-section showing stigma detail, F) anthers, G) fruit. A–F drawn from *Smith & Sanchez-Vega 7524*, G from *Hutchison et al. 5738*.

**Figure 4:**
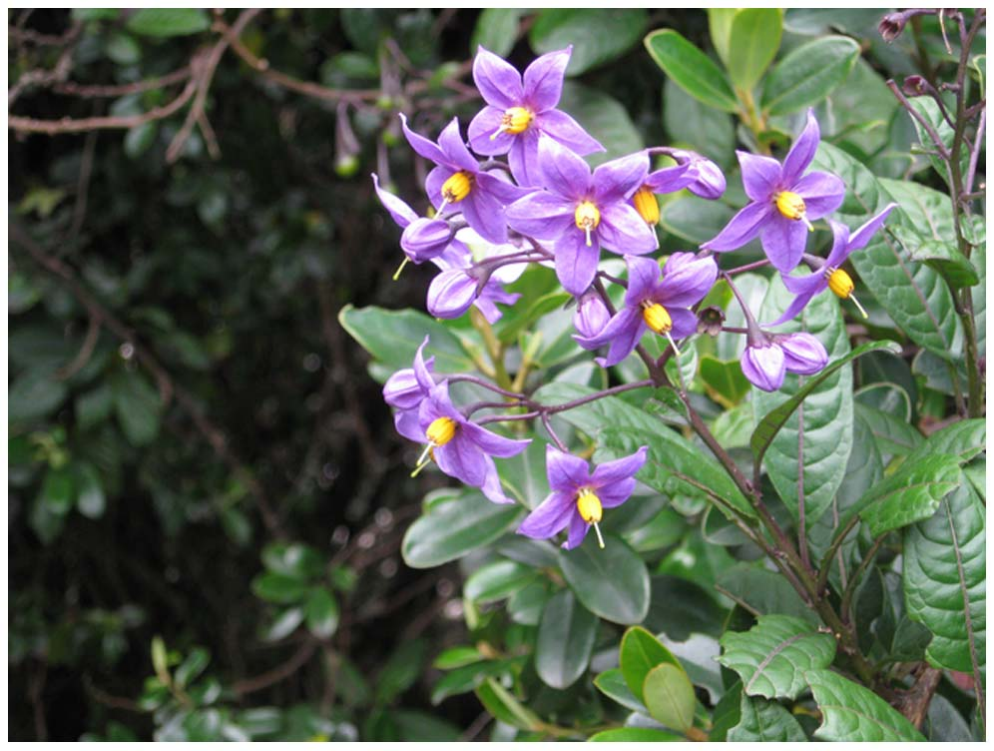
*Solanum sanchez-vegae*. Peru. La Libertad. *A. Cano s.n.* (photograph courtesy of A. Cano, USM).

Species *Solano aureo* Dunal similis, sed foliis laxe pubescentibus, floribus maioribus, stylis glabris, seminibus paucis, differt.

Woody vine or lax shrub, to 6 m; stems glabrous to sparsely pubescent with tangled loose dendritic trichomes 1–1.5 mm, these multi-celled and few branched; new growth pubescent with tangled dendritic trichomes 1–1.5 mm, occasionally almost completely glabrous; bark of older stems reddish brown, glabrescent. Sympodial units plurifoliate. Leaves simple, (2.5−)5–12× (1.3−)2.5–5 cm, narrowly elliptic, fleshy to chartaceous, the upper surfaces glabrous, the lower surfaces with loose dendritic trichomes to 1 mm long along the veins and occasionally extending to the lamina; primary veins 11–14, with a prominent intramarginal vein looping 1/3 of the way in from the margin, all veins impressed above; base acute to cuneate; margins entire, usually revolute; apex acute; petioles 1–3.5 cm, stout, glabrous to sparsely pubescent, often drying dark in herbarium specimens. Inflorescences terminal, to 15 cm long and very broad, globose in outline, branched many times from very near the base, with 50–100 flowers, glabrous to sparsely pubescent with loose dendritic trichomes; peduncle to 1 cm, the inflorescence branching very near the junction with the stem; pedicels 1.2–1.5 cm, slender, 0.5–1 mm in diameter at the base, 1–1.2 mm in diameter at the apex, spreading at anthesis, glabrous to very sparsely pubescent, articulated at the base, leaving a prominent peg from a sleeve ca. 0.5 mm long; pedicel scars irregularly spaced, often clustered, 0.5–10 mm apart. Buds globose and becoming ellipsoid, the corolla strongly exserted from the calyx tube before anthesis. Flowers all perfect, 5-merous; calyx tube 1–1.5 mm, cup-shaped but abruptly narrowing from the pedicel, the lobes 1.5–2 mm, broadly deltate and irregularly splitting, pubescent at the tips with tiny dendritic trichomes to 0.5 mm; corolla 1.9–3 cm in diameter, lilac, stellate to stellate-pentagonal, lobed ca. halfway to the base, the lobes 8–10×4–7 mm, planar or slightly campanulate at anthesis, densely papillate and pubescent at the tips and margins, the hairs extending slightly along the midvein abaxially; filament tube <0.2 mm, the free portion of the filaments 0.75–1.5 mm, glabrous and shiny; anthers 4.5–5×1.5–2 mm, yellow, sagittate at the base, poricidal at the tips, the pores lengthening to slits with age; ovary glabrous; style 7–10 mm, glabrous and shiny; stigma capitate and bifid, the surface minutely papillate. Fruit a globose berry, 1.2–1.5 cm in diameter, black, the pericarp thin, dull and matte; fruiting pedicels 1.6–2 cm long, ca. 7 mm in diameter at the apex with the apex markedly more dilated, apparently nodding in fruit; fruiting calyx lobes to 5 mm, woody, the margins paler. Seeds 4–6 per berry, 5.5–6×3–4 mm, flattened reniform, reddish brown, the surfaces minutely pitted, the testal cells round-rectangular in outline.

#### Distribution

*Solanum sanchez-vegae* occurs in cloud forest, montane forest (“ceja de selva” and “jalca”) in the Andes of northern Peru south of the Huancabamba Depression around the middle Río Marañon valley, from 2500 to 3250 m.

#### Etymology

*Solanum sanchez-vegae* is named in honor of Don Isidoro Sanchez-Vega (CPUN), whose comprehensive in-depth knowledge of the flora of northern Peru was kindly and generously shared with all who crossed his path.

#### Preliminary conservation status

*Solanum sanchez-vegae* has a relatively narrow distribution, but within that it is relatively common, and it occurs in some protected areas such as Parque Nacional Abiseo.

#### Additional specimens examined

**Peru**. **Amazonas**: Balsas-Leimebamba road, km 406, 4 Jun 1977, *Boeke 1927* (MO); Prov. Chachapoyas, 29 Jul 1991, *Mostacero et al. 2619* (MO); Prov. Chachapoyas, Atuén, Chuquibamba, 18 Jul 1995, *Quipuscoa & Bardales 187* (BM, F, MO); middle eastern slopes, near kms 411–416 of Leimebamba-Balsas road, 11 Jul 1962, *Wurdack 1314* (K, USM). **Piura**: Prov. Huancabamba, Procedencia, Cruz Blanca-Turnalina., 5 Sep 1981, *López M. & Ramírez 8926* (BM). **La Libertad**: Prov. Santiago de Chuco, Cerro La Botica, 9 Jun 1953, *López M. 1011* (US); Prov. Santiago de Chuco, 14 Jun 1984, *Sagastegui et al. 11894* (MO); Prov. Sanchez Carrión, alrededores de Huamachuco, 22 May 2001, *Sagastegui & Zapata 16535* (BM); Prov. Santiago de Chuco, 9 Jun 2001, *Sagastegui et al. 16631* (BM, F); Prov. Bolivar, junction of Quebrada Misquichilca and Quebrada Quisuar, 4 km SE Condormarca, 7°00′S, 77°00′W, 5 Jun 1986, *Young 3554* (K, USM). **San Martín**: Prov. Huallaga, valley of Río Apisoncho [ = Abiseo] 30 km above Jucusbamba, 7°55′S, 77°10′W, 2 Sep 1965, *Hamilton & Holligan 551* (K). **Cajamarca**: Prov. San Miguel, en los alrededores, Dist. Unión Agua Blanca, 9 Feb 2000, *Alvítez I. et al. 1057* (F); Prov. San Ignacio, base de Cerro Picorana, Dist. San José de Lourdes, 25 Aug 1999, *Diaz et al. 10743* (MO); Prov. San Miguel, alrededores (Agua Blanca), 5 Jul 1986, *Mostacero L. et al. 1326* (BM, F); Prov. Contumazá, sobre la ruta Salcat, Cascabamba-Pampa de la Sal, 30 Jun 1983, *Sánchez Vega 3142* (F, MO); Prov. Chachapoyas, (Agua Blanca), 12 May 1977, *Sagastegui et al. 8804* (MO); Prov. Contumazá, Contumazá-Cascabamba, 12 Jun 1981, *Sagastegui et al. 9994* (BM, MO); Prov. Cajamarca, SAIS, José Carlos de Mariátegui, km 20–40 on Sunchubamba-San Juan road, 5 Jun 1984, *Smith & Sanchez Vega 752*.

*Solanum sanchez-vegae* is a striking species, with large purple flowers and shiny rubbery leaves (Fig. 4). It has long been subsumed in the more common and widely distributed *S. aureum* Dunal, with which it is very similar. *Solanum aureum* differs from *S. sanchez-vegae* in its smaller flowers, generally denser and more congested pubescence of dendritic trichomes with many small, short branches (as opposed to loose dendritic trichomes with larger branches, see Fig. 3A) and more northerly distribution. The ranges of *S. aureum* and *S. sanchez-vegae* slightly overlap in northern Peru, but in general *S. aureum* is an Ecuadorian species. I have previously identified specimens of *S. sanchez-vegae* as *S. aligerum* Schltdl., a shrubby member of the Dulcamaroid clade with similar large, open inflorescences, but *S. aligerum* has white flowers and tufts of dendritic trichomes in the vein axils, rather than purple flowers and dendritic trichomes along the veins. *Solanum sanchez-vegae* also resembles the Venezuelan species *S. dichroandrum* Dunal, with which it shares loose pubescence and relatively large flowers; it differs from *S. dichroandrum* in its much larger (to 3 cm rather than to 2.5 cm) purple flowers (Fig. 3D, 4), glabrous style (Fig. 3E) and few-seeded berries.

**Solanum sousae** S. Knapp, sp. nov. [urn:lsid:ipni.org:names:77103636-1] Type: Mexico. Oaxaca: Mun. San Miguel Chimalapa, Cerro La Culebra, al N del Cerro Guayabitos, ca. 6 km línea recta al NO de Benito Juarez, ca. 42 km en línea recta al N de San Pedro Tapanatepec, 16°45′N, 94°11′W, 1600–1800 m, 16–18 Jul 1986, *S. Maya J. 3602* (holotype, MEXU-932219).

Figure 5.

**Figure 5:**
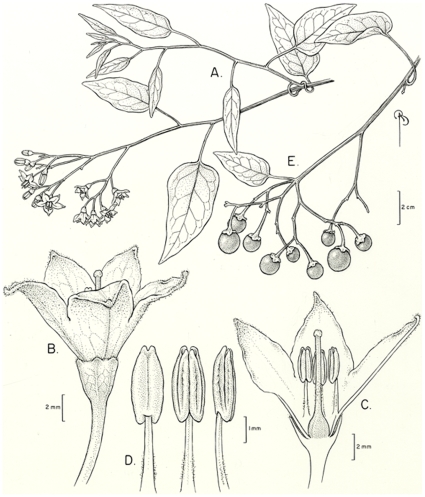
*Solanum sousae*. A) portion of stem with flowering inflorescence, B) flower, C) flower cross section, D) anthers, E) portion of stem with fruiting inflorecence. A–D drawn from *Ventura 2212*, E from *Maya J. 3938*.

Species *Solano pyrifolio* Lamarck similis, sed foliis aequaliter pubescentibus, lobis calycis minutis, antheris inaequalibus, differt.

Woody vine with trailing stems; stems sparsely pubescent with simple, uniseriate trichomes to 0.5 mm long, composed of 2–3 cells, the stems soon glabrescent; new growth densely pubescent with simple uniseriate trichomes, these whitish cream; bark of older stems pale greenish brown, glabrescent. Sympodial units plurifoliate. Leaves simple, 2.7–7(+) ×1–5 cm, narrowly ovate to elliptic, membranous, the upper surface glabrous to sparsely pubescent with simple, uniseriate trichomes on the lamina, more densely pubescent on the veins, the trichomes to 0.5 mm long, the undersurfaces almost glabrous to densely pubescent with simple, uniseriate trichomes to 0.5 mm long, these denser on the veins; primary veins 5–7 pairs, yellowish; base truncate to broadly acute; margins entire; apex acute to acuminate; petiole 1–4 cm, twining, glabrous or pubescent like the adjacent stem. Inflorescence 7–10 cm long, terminal, many times branched, more or less broadly triangular in outline, with 30–40 flowers; peduncle 3–4 cm long, pubescent like the stems; pedicels 1–1.5 cm, ca. 0.5 mm in diameter at the base, ca. 1 mm in diameter at the apex, nodding at anthesis, sparsely pubescent like the rest of the inflorescence, articulated near the base, leaving a small peg ca. 1 mm high, on the rhachis; pedicel scars spaced 0.1–0.5 cm apart, clustered near the tips of the inflorescence branches. Flowers all perfect, 5 merous; calyx tube 1.5–2 mm, conical, appearing striped from the thickened venation, the lobes <0.5 mm, mere undulations on the margin of the tube, occasionally somewhat quadrate when sinus splitting, sparsely and unevenly pubescent with simple, uniseriate trichomes to 0.5 mm; corolla 1.5–2 cm in diameter, white, stellate to pentagonal stellate, lobed 1/2 to 3/4 of the way to the base, the lobes 5–8 x ca. 4 mm, planar or slightly cupped at anthesis, densely pubescent-papillate with minute simple trichomes abaxially, glabrous adaxially; stamens with the filament tube minute, pubescent; free portion of the filaments 1.2–2 mm, very slightly unequal in some collections, pubescent near the base adaxially with tangled, simple uniseriate trichomes ca. 0.5 mm; anthers 2.5–3×1–1.5 mm, yellow, ellipsoidal, poricidal at the tips, the pores lengthening to slits with age; ovary glabrous; style 7–9 mm, pubescent with simple uniseriate trichomes <0.5 mm in the lower half; stigma capitate or somewhat bilobed, the surface densely papillate. Fruit a globose berry to 1.5 cm in diameter, green (immature?), the pericarp thin, matte; fruiting pedicels 1.5–1.7 cm, ca. 1.5 mm in diameter, woody and pendent. Seeds >50 per berry, ca. 2.5×2 mm, flattened reniform, golden brown, the testal surface minutely pitted.

#### Distribution

*Solanum sousae* is known only from southern Mexico in the states of Puebla and Oaxaca, in mesophyllous forests and oak-pine-*Liquidambar* forests on steep slopes with rich soils, from 1600–1900 m.

#### Etymology

*Solanum sousae* is named in honour of Mario Sousa Sánchez (MEXU), whose dedication to the advance of knowledge of the Mexican flora has resulted in a whole new generation of Mexican botanists.

#### Preliminary conservation status

*Solanum sousae* is known from only three widely dispersed collections, none of which falls within a protected area. It must be considered at threat, but further collecting and observation are a priority.

#### Additional specimens examined

**Mexico**. **Oaxaca**: Mpio. Santa María Chimalapa, Cerro de los Pavos, al N de Cerro Guayabitos y al O del Río Portemonedas, ca. 47 km en línea recta al N de San Pedro Tapanatepec, 16°47′N, 94°10′W, 22–23 Sep 1986, *Maya J. 3938* (MEXU). **Puebla**: Mpio. Atempan, Puente Viejo, 1900 m, 8 Jul 1986, *Ventura A. 22129* (MEXU).

*Solanum sousae* is superficially similar to *S. pyrifolium* Lam. of Hispaniola, but differs from that species in its more broadly triangular inflorescence outline (Fig. 5A), minute calyx lobes without thickened margins (Fig. 5B) and in its lack of a prominent submarginal leaf vein. The leaf pubescence of the two species is very similar, but *S. sousae* is in general more densely pubescent on the new growth and abaxial corolla surfaces. *Solanum sousae* differs from the more common and sympatric *S. dulcamaroides* Poir. in its white flowers, generally simple pubescence (versus more commonly dendritic in *S. dulcamaroides*), white rather than purple flowers, and in its anthers that are not markedly thickened and rounded abaxially.

It is likely that the juvenile leaves of *S. sousae* are pinnatifid, as are those of most other species in this group; young foliage is only very rarely collected and is often not associated with the flowering stems with simple leaves.

## Materials and Methods

### Specimens

These new species came to light during the examination of herbarium specimens during work on a monograph of the larger Dulcamaroid group (see above). Taxonomic methods follow other recent *Solanum* treatments [2], [7], [8]. All measurements are indicated in the text and were taken from preserved herbarium specimens. Specimens were examined from herbaria cited in text; herbarium acronyms follow Index Herbariorum (http://sweetgum.nybg.org/ih/).

### Nomenclature

The electronic version of this document in itself does not represent a published work according to the International Code of Botanical Nomenclature [10], and hence the new names contained in the electronic version are not effectively published under that Code from the electronic edition alone. Therefore, a separate edition of this document was produced by a method that assures numerous identical printed copies, and those copies were simultaneously distributed (on the publication date noted on the first page of this article) for the purpose of providing a public and permanent scientific record, in accordance with Article 29 of the Code. Copies of the print-only edition of this article were distributed on the publication date to botanical or generally accessible libraries of the following institutions (BM, COL, GH, HUA, K, MEXU, MO, NY, QCA, QCNE, USM). The separate print-only edition is available on request from PLoS (Public Library of Science) by sending a request to PLoS ONE, 185 Berry Street, Suite 3100, San Francisco, CA 94107, USA along with a check for $10 (to cover printing and postage) payable to “Public Library of Science”. In addition, new names contained in this work have been submitted to IPNI (http://ipni.org), from where they will be made available to the proposed Global Names Index. The IPNI LSIDs (Life Science Identifiers) can be resolved and the associated information viewed through any standard web browser by appending the LSID contained in this publication to the prefix http://ipni.org/.

The online version of this work is archived and available from the following digital repositories: PubMedCentral (www.pubmedcentral.nih.gov/) and Solanaceae Source: a web resource for the nightshade family (http://www.solanaceaesource.org).
